# Ethics and Regulation of Human Brain Organoid Research: Recommendations from the Asia Pacific Neuroethics Working Group

**DOI:** 10.1007/s41649-025-00398-6

**Published:** 2026-04-29

**Authors:** Shu Ishida, Brett J. Kagan, Masanori Kataoka, Julian Koplin, Sebastian Porsdam Mann, Jonathan Lewis, Heather Browning, Alexandre Erler, Faisal Feroz, Tamami Fukushi, Søren Holm, Masatoshi Kokubo, Stephen Latham, Andrea Lavazza, Ilhak Lee, Tsung-Ling Lee, David Lyreskog, Jerry Menikoff, Takuya Niikawa, Naoya Nagaishi, Eisuke Nakazawa, Serene Ong, Koji Ota, Christopher Register, Walter Veit, Ji Hyun Yang, Shang Long Yeo, Tsutomu Sawai, Julian Savulescu, Brian D. Earp

**Affiliations:** 1 Uehiro Division for Applied Ethics, Graduate School of Humanities and Social Sciences, Hiroshima University, Higashihiroshima, Japan; 2 Cortical Labs, Melbourne, Australia; 3 Department of Biochemistry and Pharmacology, School of Biomedical Sciences, University of Melbourne, Melbourne, Australia; 4 Monash Bioethics Centre, Faculty of Arts, University of Melbourne, Melbourne, Australia; 5 Center for Advanced Studies in Bioscience Innovation Law, Faculty of Law, University of Copenhagen, Copenhagen, Denmark; 6 Faculty of Law, University of Oxford, Oxford, UK; 7 Centre for Biomedical Ethics, Yong Loo Lin School of Medicine, National University of Singapore, Singapore; 8 Department of Philosophy, University of Southampton, Southampton, UK; 9 Institute of Philosophy of Mind and Cognition, National Yang Ming Chiao Tung University, Hsinchu, Taiwan; 10 Faculty of Human Welfare, Tokyo Online University, Tokyo, Japan; 11 Centre for Social Ethics and Policy, Department of Law, The University of Manchester, Manchester, UK; 12 Centre for Medical Ethics, HELSAM, University of Oslo, Oslo, Norway; 13 Interfaculty Initiative in Information Studies, Graduate School of Interdisciplinary Information Studies, University of Tokyo, Tokyo, Japan; 14 Yale Interdisciplinary Center for Bioethics, Yale University, New Haven, CT USA; 15 Department of Literary, Linguistic, and Philosophical Studies, Pegaso University, Naples, Italy; 16 Department of Medical Humanities and Social Sciences, College of Medicine, Yonsei University, Seoul, Korea; 17 Graduate Institute of Health and Biotechnology Law, Taipei Medical University, Taipei, Taiwan; 18 NEUROSEC, Department of Psychiatry, University of Oxford, Oxford, UK; 19 Faculty of Law, National University of Singapore, Singapore; 20 Graduate School of Humanities, Kobe University, Kobe, Japan; 21 Graduate School of Law, Hitotsubashi University, Kunitachi, Japan; 22 Department of Biomedical Ethics, Faculty of Medicine, University of Tokyo, Tokyo, Japan; 23 Institute of Humanities and Social Sciences, University of Tsukuba, Tsukuba, Japan; 24 Uehiro Oxford Institute, University of Oxford, Oxford, UK; 25 Department of Philosophy, University of Reading, Reading, UK; 26 Graduate School of Humanities and Social Sciences, Hiroshima University, Higashihiroshima, Japan; 27 Institute for the Advanced Study of Human Biology (ASHBi), Kyoto University, Kyoto, Japan; 28 Biomedical Ethics Research Group, Murdoch Children’s Research Institute, Melbourne, Australia; 29 Melbourne Law School, The University of Melbourne, Melbourne, Australia

**Keywords:** Human brain organoids, Neuroethics, Moral status, Regulation, Consent, Science communication

## Abstract

Human brain organoids (HBOs) are three-dimensional structures derived from human stem cells that model aspects of brain development and function, offering potentially unprecedented opportunities for studying neurological disorders and for developing treatments. This consensus paper presents recommendations from the Asia Pacific Neuroethics Working Group, developed through interdisciplinary collaboration among scientists, bioethicists, philosophers, and legal scholars who convened in Singapore in November 2024. We provide a comprehensive analysis of the ethical, legal, and sociocultural dimensions of HBO research, addressing both current realities and future possibilities. The paper examines key ethical considerations, including the potential moral status of HBOs, particularly regarding sentience and consciousness, while identifying and dispelling common misconceptions and “ethical red herrings” arising from sensationalized portrayals. We analyze consent frameworks for cell donation, privacy concerns, dual-use risks, and questions of distributive justice. Legal challenges are explored, including the categorical ambiguity of HBOs within existing regulatory frameworks, intellectual property issues, and cross-border inconsistencies in standards. Sociocultural perspectives emphasize the importance of public understanding, cross-cultural engagement, and empirical research on diverse community attitudes toward HBO research. In our recommendations, we advocate for evidence-based ethical discussions, anticipatory frameworks addressing potential future developments, contextualized analysis comparing HBOs to related experimental models, robust informed consent processes, proportionate responses to consciousness concerns, development of adaptive regulatory frameworks, responsible science communication to manage public expectations, and sustained interdisciplinary collaboration. We emphasize a balanced approach that promotes scientific innovation while maintaining rigorous ethical oversight, recognizing HBOs’ significant potential for advancing neuroscience and medicine. This represents the first comprehensive ethical framework for HBO research from the Asia Pacific region, helping to establish foundational principles for responsible development of this rapidly advancing field.

## Introduction

Human brain organoids (HBOs) are three-dimensional biological structures grown in vitro from human stem cells, which, through a combination of self-organization and researcher intervention, take on some of the functional and structural properties of the human brain. Rather than being fully functional human brains, however, they serve as simplified models — albeit of varying levels of complexity and maturity depending on research aims and technical capacity — of certain aspects of brain structure or function. The purpose of creating HBOs is to facilitate the study of neural development, neurological disease, and ultimately potential treatments, going beyond what has been possible with traditional cell culture techniques. It is hoped that, in addition to yielding scientific insights, this research will enable significant future benefits for human health and well-being (for a review of existing knowledge and benefits, see Eichmüller and Knoblich 2022).

Alongside rapid advances in the scientific and technical aspects of this research, reviewed briefly in Sect. “Science of HBOs: A Brief Introduction” below, there is growing awareness of the need to consider social, ethical, and legal issues while engaging the interdisciplinary expertise of diverse stakeholders and taking an international perspective. Here, we present a high-level overview of the current scientific and ethicolegal dimensions of brain organoid research representing the consensus view of the Asia Pacific Neuroethics Working Group. The authors consist of a core group of scientists, bioethicists, philosophers, and legal scholars who gathered for an intensive 2-day professional meeting on 11–12 November 2024 at the National University of Singapore and an extended network of close collaborators with relevant interdisciplinary expertise (see Appendix for details).

While a diversity of opinions was observed among Working Group members, we emphasize areas of broad agreement and also note the unresolved issues. We begin with a brief technical section on HBOs, including a snapshot of the latest scientific advances (Sect. “Science of HBOs: A Brief Introduction”), followed by an overview of the main reasons why research into, and potential applications of, HBOs are considered ethically and potentially legally significant. We identify what we regard as problems around hype in the literature, as well as “ethical red herrings” (ostensible ethical issues raised by HBOs that are based on misunderstandings or errors in reasoning). This is followed by substantive ethical, legal, and psychosocial considerations raised by HBOs (Sects. “Putative Moral Relevance of HBOs”–“Sociocultural Considerations”), policy recommendations (Sect. “Practical and Policy Recommendations”), and working conclusions and future directions (Sect. “Interdisciplinary and Cross-Cultural Engagement”).

## Science of HBOs: A Brief Introduction

HBOs first arose as a method to study neurodevelopment, with a focus on understanding the different cell types that arose in different cortical layers. Interest quickly grew to include disease modeling, pharmacological interventions, and even interspecies differences (Pollen et al. 2019; Kim et al. 2020). Despite these exciting avenues of research, the development of HBOs did not involve a radical departure from more established cell culture techniques (Kagan et al. 2025). This is important to note as, in some discussions, HBOs are positioned as a totally novel culturing technique that may require distinct ethical considerations; when in practice, significant overlap with other methods exists. Differentiation of neural cell cultures, whether monolayers or multi-layer organoids, typically can follow either a direct differentiation or an ontogenetic differentiation approach. Direct differentiation involves upregulating key genes observed in the desired post-mitotic cell type to facilitate a rapid differentiation from stem cell to neural subtype (Hulme et al. 2022). The ontogenetic approach involves using small signalling molecules that are varied in concentration and time of administration to direct a more physiologically relevant differentiation process (Hu et al. 2010). Both methods have advantages and disadvantages in terms of protocol speed, variability, and physiological relevance.

The key difference in whether a method generates neural monolayers versus HBOs is if cells are allowed to generate spherical-type structures. To achieve this, the typical approach is to use an enzyme to detach adherent cells from their culturing plate and briefly centrifuge the cell suspension in low-attachment plates. Cells will attach to each other and, provided they are maintained under appropriate conditions — usually on a moving shaker that does not allow attachment to another surface — the culture will develop into HBOs. HBOs can have certain advantages over some monolayers, in some cases showing more mature cells or more complex electrophysiological activity (Fitzgerald et al. 2024). However, more complex electrophysiological dynamics can also be achieved with different treatments of monolayers (Yamamoto et al. 2018). Scientifically, therefore, the differences exist on a spectrum.

The starkest differences (aside from the catchiness of terminology) are perhaps the appearance of the cell cultures, as HBOs can seem more visually impressive with a robust 3D structure reminiscent of “mini-brains”. Yet, this terminology is misleading and risks underlying inferences about HBO properties, functions, capacities, and moral standing that are simply not supported by the current state of the research (Gaillard and Botbol-Baum 2022). While HBOs can model isolated regions of the human brain remarkably well, current methods for generating them do not result in an in vitro organoid that matures beyond the equivalent of parts of an early prenatal brain. Although organoid researchers have claimed that it is conceptually possible to combine multiple cell types or lineages (i.e. cells from different regions of the brain) in 3D culture or co-culture to produce neural assembloids (i.e. an assembly of several HBOs modelling different brain regions), there are major limitations to HBO development in terms of modelling the entirety of the human brain — e.g. the problems of oxygen and nutrient diffusion and waste removal, the absence of a peripheral nervous system, and the problems of modelling interactions between different parts of the brain and understanding the neural activity of HBOs (see, e.g. Cakir et al. 2019; Chen et al. 2019; Andrews and Kriegstein 2022; LaMontagne et al. 2022).

Despite these limitations, HBOs remain scientifically useful. However, awareness of the existence and implications of these limitations, as well as the scientific continuity between traditional (monolayer) and multi-layer (HBO) cell culture methods, is critical to prevent mistakenly ascribing properties to HBOs that might — whether rightly or wrongly — seem to have outsized moral relevance. In other words, to have a clear-minded discussion about the potential moral status of HBOs, if any, it is necessary first to have a scientifically accurate picture of the empirical properties they do and do not have and hence the capacities it is reasonable to infer they can or cannot support. That being said, current differences are not always clear and so far are a matter of quantitative changes on a spectrum, rather than qualitative or categorical step changes that require entirely different perspectives.

## Putative Moral Relevance of HBOs

### General Ethical Issues

Some ethical concerns raised by HBO research are broadly shared with other developments in neuroscience and neurotechnology. These include questions about how to ensure valid consent (or to enable withdrawal of consent) of potential donors of biological material, misuse of samples or research findings, privacy concerns, and a need to ensure equity in the distribution of risks and benefits, among other issues. We first briefly canvass some of these broader issues before turning to the properties of brain organoids that are widely thought to raise distinctive ethical concerns.

First, as with other stem cell science, HBO research demands careful attention to protecting the interests of research participants, typically through informed consent, especially with regard to unforeseen future applications of donated human-derived cells (Barnes et al. 2025). In the context of HBO research, a key issue arises around whether cells should continue to be used under broad consent (i.e. where research is conducted for diverse purposes within a general framework that may include HBO research), specific consent (explicitly specifying the intended research purpose), or some other model of consent like dynamic consent and consent for governance frameworks (Lewis and Holm 2022, 2024, 17–21; Kataoka et al. 2024b). Linked to the issue of consent is that of participant withdrawal. This class of ethical issues includes the conditions under which a cell or tissue donor should be permitted to withdraw their cells, the extent to which cell donors should be allowed to withdraw their cells (especially if turned into a cell line), and whether donors should be permitted to withdraw the HBOs that have been derived from those cells (Lewis and Holm 2024, 25–27).

Second, privacy is also a significant ethical consideration in virtually all scientific research involving human beings. In the specific context of HBO research (like other scientific fields utilizing human cells), however, one commonly raised concern has to do with genetic or epigenetic information specific to the donor, which might risk violating the donor’s privacy during scientific research (Lewis and Holm 2022). If research reveals traits linked to cognition, mental health, or predispositions for neurological disorders, for example, there is a risk of this information being misused, leading to breaches of privacy or even discrimination against donors. Although there are some complications as to the precise relationship between HBOs and privacy issues (Kataoka et al. 2025a), the intimate connection between HBOs and their human origins could make such risks more acute than, say, neurotechnologies that do not rely on donor-derived material.

A related ethical issue concerns the handling of incidental findings and sensitive information derivable from genetic information. As demonstrated in other fields like clinical proteomics, biological samples can reveal unexpected but potentially significant health information about donors (Geyer et al. 2021; Mundt et al. 2023; Porsdam Mann et al. 2021). With HBOs, such findings might include previously unknown neurological predispositions or conditions. These findings may be actionable (where preventive or therapeutic interventions are available) or non-actionable (where no current treatments exist), each potentially requiring different protocols for management and disclosure. The possibility of such findings should be addressed during the informed consent process, with clear procedures established for if and how information would be returned to donors. Again, incidental findings and other sensitive information raise common ethical questions applicable to many scientific domains including but not limited to HBO research.

Third, research involving the transplantation of HBOs into animals has raised concerns about blurring the line between humans and non-human animals, leading to disruption of the established ethical and legal frameworks (Robert and Baylis 2003, 2021). How the blurring of this boundary is considered morally significant varies across cultures (Crane et al. 2020), which should be reflected in the local governance of the transplantation of HBOs (see also Sect. “Sociocultural Considerations”). In addition, the welfare and potential enhancement of chimeric animals are important ethical issues in the xenotransplantation of HBOs as well (Chen et al. 2019; Birch and Browning 2021; Kataoka et al. 2023a; Harary et al. 2023; Erler 2024). These ethical considerations relating to animal transplantation studies on HBOs are shared by other human-animal chimera research.

Fourth, it is necessary to be aware of the broader dual-use potential of HBO research. Beyond the issue of privacy violations noted above, the possible generation of neurodata linked to cognition, emotion, and/or behavior — either at individual or population levels — may heighten risks of misuse in areas such as problematic commercialization (Boers et al. 2019), manipulative marketing practices, law enforcement, or surveillance. In addition, HBOs themselves can be used in toxicity testing and integrated with machinery, which may lead to harmful or even military uses (Rinaldi and Colotti 2019; Mollaki 2021). These worries underscore the importance of rigorous oversight and clear boundaries in HBO research to mitigate risks not only to donors but to broader societal interests.

Finally, HBO research also faces the ethical question of distributive justice. As in other fields of biomedical research, one may be concerned about the commercialization and patentability of entities produced using donated biological materials and HBO cultivation techniques (see, e.g. Boers et al. 2019; Lewis and Holm 2024, 40–47). Should the profits gained through the research benefit only certain for-profit companies, or should they be distributed among all stakeholders involved in the process? Or should proprietary elements be minimized or even eliminated to ensure research freedom and maximum accessibility of its outcomes for current and future patients (see Sect. “Legal Considerations” below) (cf. Molnár-Gábor 2019; Porsdam Mann et al. 2024a)? In addition, potentially expensive HBO-based therapies (when they are ready in the future) may risk exacerbating existing inequalities without, say, proactive measures like egalitarian healthcare coverage and fair accessibility.

### Possibly Distinctive Ethical Issues

Besides these ethical issues that are (more or less) applicable to other scientific fields, there are also more specific reasons why HBO research is thought to raise distinctive ethical considerations. These stem from the biological nature of HBOs and their future potential, increased neural complexity, and possible associated functionality. Some of these issues will relate to the current nature and function of HBOs, while others may relate to the capacities HBOs may have in the future. Unlike traditional neurotechnologies, these entities are derived from human stem cells and can develop neural networks resembling aspects of the human brain. They can exhibit spontaneous electrical activity or rudimentary responses to stimuli, raising questions about their ability — particularly as research develops — to support sentience, consciousness, or other mental properties widely regarded as morally significant (Kataoka et al. 2025c). As we will discuss in the following section, some of these questions may be “ethical red herrings” rather than genuine moral concerns. Nevertheless, as the complexity of HBOs increases, they could hypothetically acquire certain psychological properties compelling us to consider how they should be treated and whether they could ever deserve rights or protections per se.

In addition to the well-known issue of blurring the boundary between humans and non-human animals mentioned above, HBO research also has the potential to shake other possibly ethically important boundaries (Boyle 2024). A recent study, for example, created a single HBO from the cells of multiple donors, which provides an unfamiliar entity of nervous tissues consisting of nerve cells of different individuals (Antón-Bolaños et al. 2024). Furthermore, research is rapidly advancing on combining mono-layered human nerve cells and HBOs with computers (Kagan et al. 2022a, b; Smirnova et al. 2023), which already sparks ethical debates (Kagan et al. 2023a, b; Kataoka et al. 2024a). The potential of HBOs for open-ended development and integration with artificial intelligence (AI) would complicate matters further. Thus, HBOs have the potential to result in novel hybrid entities with unpredictable capabilities (Kagan 2025). Existing ethical guidelines may be ill-equipped to address these novel entities, potentially requiring new or revised frameworks to navigate the emerging questions around rights, protections, and permissible uses of these human brain cell-derived entities.

## Concerns Around Communication

### Misunderstandings and Hype

In this viewpoint article, we present the consensus perspective of the Asia Pacific Neuroethics Working Group on the social, ethical, and legal considerations raised by HBO research, as it emerged from the symposium mentioned previously. However, we must first address potential ethical red herrings, some of which likely derive from the hype around the existing science, philosophical errors in reasoning, or simple misunderstandings (Gaillard and Botbol-Baum 2022; Hofmann et al. 2022; Ravn et al. 2023; Vogt et al. 2023).

The ethical discourse surrounding HBOs is often clouded by sensationalism and misunderstandings, leading to concerns that, while dramatic, are currently misplaced (Presley et al. 2022).^1^ For instance, the idea that HBOs are “mini brains” capable of thought, memory, or self-consciousness has captivated the public imagination but misrepresents their current capabilities (International Society for Stem Cell Research 2021; Gaillard and Botbol-Baum 2022; Kataoka et al. 2023b; Ravn et al. 2023). While these structures can mimic certain aspects of brain tissue, they lack the degree of structural complexity, functional connectivity, and systemic integration characteristic of an adult human brain (Urrestizala-Arenaza et al. 2024).

Similarly, fears about an imminent emergence of consciousness (see Sect. “Moral Status of HBOs” below for details) in HBOs would exaggerate their developmental potential at this stage of research. While it is wise to anticipate potential future ethical challenges, the near-term risks of HBOs achieving anything resembling human-level consciousness or self-consciousness are significantly lower than often suggested in media articles. As scientific progress is naturally iterative, metrics that link with these morally relevant traits can be measured and assessed across increasing levels of complexity and functionality, which could function as an ethical safeguard (Kagan et al. 2022a, b). However, this requires active exploration of these traits to be conducted as new HBO protocols are developed, as well as an anticipatory approach to be adopted as previously described (Kagan et al. 2023a, b). Particular attention may be needed, for example, for HBOs designed to replicate human evaluations via the neuroeconomic pathways of reward that would be necessary for subjective suffering/pain (Browning and Veit 2023a).

Other concerns arise from equally speculative narratives. Some may worry that HBOs replicate the minds or personalities of their donors, invoking anxieties about “psychological cloning” and human identity. Although HBOs can genetically reflect their donors, represent certain structural and functional aspects of an early prenatal human brain, and be of symbolic and moral value to their donors (Lewis and Holm 2022), they are far too simple to reflect anything like a donor’s cognitive traits or personality (Kataoka et al. 2025a). While it is important to remain vigilant as science advances, these red herrings and hype surrounding HBOs — especially when put as if they were near-future problems — risk diverting attention from genuine ethical questions and the need for thoughtful, evidence-based regulation of this emerging field (International Society for Stem Cell Research 2021).

That said, it is not wise to just disregard these popular concerns simply because they are speculative or lack a scientific basis. They may represent an expression of a lack of trust in the scientific community, which in turn may undermine societal support, regulatory cooperation, and funding decisions. Sincere and transparent communication regarding current regulatory constraints and limitations on the scientific knowledge concerning HBOs is essential for (re)building trust between researchers and the public, particularly given the complexity and novelty of HBO research (International Society for Stem Cell Research 2021).

### Nomenclature

One of the obstacles to such clear communication is the fact that there is no firm agreement on important terms related to HBOs even among researchers within or between fields (Kagan et al. 2024b), a fact which also impacts the present paper. The discrepancies range widely, from the very question of what HBOs should be called (Pașca et al. 2022), to ethically important terms such as ‘intelligence’ or ‘consciousness’ (Rommelfanger et al. 2023; Kagan et al. 2023b; Pereira et al. 2023). Responsible use of terminology takes into account not only scientific accuracy but also its impact on normative discussions (Salles and Farisco 2024). Ensuring appropriate means of scientific communication by combining multidisciplinary coordination to refine and normalize key terms for broad usage (Kagan et al. 2024b), with research on the influence of languages on citizens’ moral judgements (Feroz et al. 2025), is urgently needed.

## Ethical Considerations

### Moral Status of HBOs

For an entity to have moral status means that it matters morally for its own sake, rather than for the sake of others who care for it or otherwise are affected by its instrumental effects.^2^ One of the most commonly accepted grounds for moral status — and the one that the debate around the potential moral status of HBOs often centres on — is sentience, defined here as the capacity for consciously experiencing positively and negatively valenced subjective states.^3^ Since there was broad consensus among the authors that sentience is an important basis for moral status, the following discussion is written primarily from this perspective. This does not mean, however, that other possible grounds are unimportant, including other forms of consciousness (see discussion below), or other complex cognitive capacities that might be required for decision-making or planning, as well as relational properties (see discussion in Sect. “Extrinsic and Relational Moral Concerns”).

#### Sentience

There is widespread agreement that sentience, as defined here (particularly the capacity to suffer), is sufficient — if not necessary — for moral status (see, e.g. Warren 1997; Levy and Savulescu 2009; Lee 2022; Singer 2024; Dung 2024; for criticism, see Ishida and Sawai 2024). Discussions on the moral status of HBOs therefore typically focus on whether these lab-grown neural structures are currently, or will plausibly one day be, sentient (see, e.g. Farahany et al. 2018; Lavazza and Massimini 2018; Koplin and Savulescu 2019; Sawai et al. 2019, 2022; Hyun et al. 2020; Lavazza 2020; Bollinger et al. 2021; Gaillard and Botbol-Baum 2022; Holm and Lewis 2022; Niikawa et al. 2022; Browning and Veit 2023a; Zilio and Lavazza 2023; Lewis and Holm 2024, 28–33).

While current HBOs are very likely to lack the complexity required for sentience (International Society for Stem Cell Research 2021), their potential to develop it raises questions about their treatment. As a very recent attempt to model pain-related sensory pathways using assembloids illustrates (Kim et al. 2025), proactive considerations about potential sentience are increasingly important, not just mere speculation.

Some argue that if HBOs achieve a level of functional organization that could plausibly support sentience, they might require protection (Birch and Browning 2021; Browning and Veit 2023a; Birch 2024). Others contend that subjective experience in general depends not only on neural activity but also on factors like complex functional interactions with the external environment, which current HBOs in vitro fundamentally lack (Hyun et al. 2022). Partly due to the uncertainties around neural correlates of sentience, current debates could not avoid speculative elements completely, but the situation underscores the importance of proactive ethical frameworks and anticipatory bioethics work to address future developments in this rapidly advancing field (Brey 2012).

At the aforesaid symposium, participants generally agreed that if HBOs were found to have the capacity to experience pain, this would warrant consideration of some moral status. This wording avoids assuming consensus on the definition of sentience and does not imply the current existence of sentient HBOs. If they have such a capacity, all else being equal, there would be ways they should or should not be treated in and of themselves: for example, by not being subjected to wanton or otherwise needless pain. In short, if brain organoids were, in the future, to reach such a threshold, we would then need to consider what their interests consist of, and take these into consideration in our treatment of them (Browning and Veit 2023b). As described below, this is no easy task.

#### Other Forms of Consciousness

Sentience, as we define it here, is a form of consciousness. As suggested above, “consciousness” is a term with significant semantic ambiguity, which has confused the debate surrounding the moral status of HBOs (Kagan et al. 2024a, b). To navigate this debate, it is crucial to differentiate between the various definitions and dimensions of consciousness and consider which might plausibly apply to HBOs (see Hyun et al. 2020; Holm and Lewis 2022; Lewis and Holm 2024, 28–33; Kataoka et al. 2025c).

One philosophically prevalent form of consciousness is phenomenal consciousness, or subjective experience in general — for something to have phenomenal consciousness means that there is something it is like to be that thing (Nagel 1974; Block 1995). While valenced experiences produced by sentience are a type of phenomenal consciousness, there are non-valenced experiences, like purely visual or auditory experiences. Unlike sentience, it is controversial whether entities that can possess only non-valenced experiences have moral status (Levy and Savulescu 2009; Niikawa 2018; Shepherd 2018). In addition, even if advanced HBOs possess phenomenal consciousness, its quality is likely to be very different from that of humans. For, they are unlikely to be structurally identical to a human brain, and they will have a peculiar mode of interaction with their environment, especially when integrated with machines or computers. This possibility of alien forms of phenomenal consciousness would further complicate ethical evaluation (Gaillard and Botbol-Baum 2022; Diner 2023).

Another important form of consciousness is access consciousness, which refers to the ability to use information in cognitive processes, possibly even without phenomenal consciousness (Block 1995). Some consider access consciousness to be the basis of moral status (Levy and Savulescu 2009). However, as it presupposes or constitutes fairly complex cognitive capacities like decision-making and planning, it is doubtful whether near-future HBOs in vitro can possess this form of consciousness. Still, particular caution would be required from this perspective when conducting research on integrating HBOs with computers. Similarly, self-consciousness, the ability to recognize oneself as a distinct entity, is difficult to realize in HBOs due to the lack of plausible natural correlates such as a default mode network or prefrontal cortex activity (Davey and Harrison 2018), although this form of consciousness is often linked to moral status.

In contrast, functional consciousness (goal-directed activity) and minimal consciousness (basic responsiveness to stimuli) are more likely to be relevant to current HBO research. A hybrid system combining cultured human nerve cells and machines appears to already be demonstrating goal-directed activity (Kagan et al. 2022a, b), and minimal consciousness could manifest as rudimentary responses to stimuli, such as neural firing. However, both forms are controversial in terms of their moral significance (Levy and Savulescu 2009; Kataoka et al. 2024a), and even if they do confer moral status, that status would not be equivalent to that of sentient beings. In particular, special caution is needed here not to confuse the response to nociceptive stimulus with the phenomenal consciousness of pain (Salomons and Iannetti 2022).

#### General Policy for Decision-Making

While certain forms of consciousness, especially sentience, do confer some moral status, even an advanced HBO is unlikely to have full moral status equivalent to humans in the foreseeable future. Therefore, a complete prohibition on research using HBOs (or sentient organisms in general) is not necessarily the appropriate solution even if morally relevant traits do arise. While we share the view that it is essential to give due consideration to pain and suffering and to base relevant regulations on this principle, harmful research is already undertaken with entities that are either certainly sentient (e.g. research mice) or plausible candidates for sentience (e.g. bees; see Gibbons et al. 2022) under reasonable justification. So, it would be inconsistent to think that sentience by itself justifies prohibiting research with potentially sentient brain organoids while accepting that sentient animals are used in research (Kataoka et al. 2023b).^4^ For consistency, if sentience is sufficient to underwrite some degree of moral status, then the question of how a sentient HBO should be used in a research context could, in principle, be considered within the context of the ongoing ethical debates concerning non-human animal research and animal rights more generally (Koplin and Savulescu 2019; Kataoka et al. 2023a; Lewis and Holm 2024). Importantly, a stance that were to prohibit HBO research on the grounds of the remote possibility of sentience could also lead to perverse outcomes by preventing the use of brain organoids in cases where they might replace research animals that are likely to experience far greater suffering than the organoids (Kagan et al. 2023a, b; Kataoka et al. 2024b; Koplin 2024). Therefore, it is crucial to keep in mind practical and proportionate responses to the potential for suffering (Birch 2024). In line with regulation of research using other sentient organisms, this should include consideration of the possible welfare interests of future sentient organoids (Holm and Lewis 2022; Browning and Veit 2023b).

#### Uncertainties in Moral Evaluation and Detection

As we have suggested, while the moral significance of sentience is well-established, that of other various forms of consciousness is still under debate and thus uncertain. Moreover, potential means of detecting various forms of consciousness (including sentience) in HBOs are not well-established. Indeed, competing theories of consciousness such as Integrated Information Theory and Global Neuronal Workspace Theory have completely divergent implications for the prospect of phenomenal consciousness (or its signs or correlates) in HBOs (Lavazza and Massimini 2018). Although the science of consciousness has been developing rapidly in recent years, uncertainty will remain for some time regarding the detection of phenomenal consciousness in HBOs (Bayne et al. 2020). In brief, current and near-future decision making regarding HBO research must face various uncertainties: whether HBOs have a form of consciousness, how to detect that form of consciousness (Kagan et al. 2024a, b), and how morally important that form of consciousness is.

#### Guiding Principles

For decision-making under such uncertain circumstances, we can appeal to some guiding principles. As advances in bioengineering increase the complexity of organoids, some versions of the precautionary principle suggest that researchers should treat organoids that are plausible “sentience candidates” as meriting protections, despite continuing uncertainty and disagreement concerning whether they are, in fact, sentient (Sebo 2018; Koplin and Savulescu 2019; Birch 2024). This might involve abstaining from research with sentience candidates, or (perhaps more reasonably) subjecting research on potentially sentient brain organoids to forms of research review and oversight similar to that currently required for research using sentient animals, with the possible harms to organoids weighed against the necessity and anticipated benefits of the research. However, neither restriction should apply to the many kinds of brain organoids for which there are no plausible indicators of sentience. While the most radical versions of the precautionary principle might call for banning organoid research regardless of the social costs, more moderate versions — such as that defended by Birch (2024) — can give a central place to the idea of proportionality.

Proportionality allows the examination of the risks of attributing a moral status to a HBO that in fact has none in terms of four key conditions: (1) the importance of the objective of the research, (2) the relevance of the means to achieve it, (3) whether the chosen approach represents the most favourable option, and (4) that the means used are not excessive in relation to the goal (Hermerén 2012). This principle is particularly important given that preventing all research with potential sentience candidates could block valuable scientific progress while in fact leading to greater overall suffering (Żuradzki 2021), not only by preventing or impeding the discovery of potential treatments for neurological disorders that cause suffering in humans, but also by requiring the use of alternative research methods such as animal models, where the certainty and/or magnitude of causing harm is likely to be greater.

The modest version of the precautionary principle with proportionality has already proven useful in evaluating similar conceptual challenges, such as in chimeric animal research involving human neural tissue, where researchers must also navigate questions of moral status uncertainty and potential consciousness (Porsdam Mann et al. 2019, 2025). Applied to HBOs, proportionality suggests that the closer research brings HBOs to sentience or other morally significant forms of consciousness, the stronger the justification must be for conducting such research, rather than imposing outright prohibition. Researchers would need to demonstrate not only the significance of their objectives but also that HBOs are necessary and appropriate tools for achieving these aims and that no less morally problematic alternatives exist.

### Extrinsic and Relational Moral Concerns

Concerns about the moral status of HBOs need not depend entirely on the intrinsic properties of the organoids themselves, such as whether they possess some sort of consciousness. Instead, according to some views, the moral status of an entity has to do with the relationships between it and other entities, or how it is situated in a certain sort of system (Warren 1997).^5^ In some Indigenous or Eastern philosophical traditions, moral worth is understood less in terms of individualistic traits and more in terms of an entity’s role within a web of relationships or its capacity to participate in a harmonious balance (Bai 2009; Graness 2019; Peterson 2019).^6^ Indeed, even within Western legal traditions, rights and protections can be extended to non-human entities — corporations, for instance, are granted legal personhood and certain associated rights based on their social and economic relationships rather than any intrinsic properties (Kataoka et al. 2023c). From this perspective, the determination of the moral status of HBOs requires a careful examination of their relationships with human society, including cell donors (van Till et al. 2023). It has also been observed that some citizens feel a special personal connection to HBOs derived from their own cells (Bollinger et al. 2021). Such findings further highlight the need for in-depth engagement with relational aspects of the moral status of HBOs, building on non-Western philosophical insights as well as the emerging trend of relational ethics within Western philosophy. We will return to the importance of cross-cultural ethical engagement later in Sects. “Practical and Policy Recommendations” and “Interdisciplinary and Cross-Cultural Engagement”.

Furthermore, relational factors could be ethically important independently of their implications for the moral status of HBOs. For example, our treatment of HBOs might reflect on or affect us as human moral agents. Kant, for example, famously argued that while non-human animals lack moral status in themselves, it is still wrong to treat them abusively because such behaviour could corrupt our character, foster callousness, or encourage a disposition toward cruelty that might ultimately harm our relationships with other humans (Kant 1797). In a similar vein, some might argue that mistreating (or appearing to mistreat) brain organoids — regardless of their moral status — might degrade our moral sensibilities or normalize practices that devalue life, sentience, or the broader responsibilities associated with scientific inquiry (Lavazza and Reichlin 2023).

At this point, it is necessary to revisit the issue of communication concerning HBOs. If ordinary people come to believe, rightly or wrongly, that organoids are sentient or capable of suffering, then treating them in ways perceived as cruel or exploitative could erode public trust in science. This relational dynamics creates ethical stakes even if the organoids themselves are not sentient. Furthermore, the symbolic significance of creating and experimenting on entities that resemble aspects of human biology could provoke broader anxieties about crossing ethical boundaries in science or “playing God.” These perceptions, even if scientifically unfounded, highlight again the importance of public engagement and transparent communication about the nature and capabilities of organoids (see, e.g. International Society for Stem Cell Research 2021), a matter to which we return in a later section. Addressing such relational issues is crucial not only for fostering ethical research practices but also for maintaining a respectful and reflective relationship between science and society.

## Legal Considerations

Beyond ethical considerations, there are also distinctive legal challenges posed by HBO research that will need to be addressed (Kataoka et al. 2024c; Lewis and Holm 2024). A fundamental challenge stems from the fact that existing legal frameworks were designed around clear categories — human subjects, animal subjects, human tissue, or inanimate research materials — yet HBOs do not fit neatly into any of these (Boers et al. 2019; Gaillard et al. 2025). This categorical ambiguity has direct practical implications (see, e.g. Lewis and Holm 2024). If HBOs were to be classified as human subjects, this would trigger extensive human rights protections and research restrictions under international human rights law. If categorized as human tissue, they would fall under different regulatory frameworks governing tissue donation and research. If treated as animal research models, yet another set of regulations would apply. The potential development of properties like sentience or other forms of consciousness further complicates this categorization, as existing laws generally do not contemplate the emergence of such capacities in laboratory-created tissue (cf. Lavazza and Pizzetti 2020; Kataoka et al. 2023c).

This legal gray area may necessitate new definitions and protections and/or reconceptualization of relevant legal rights and interests. Similarly, the use of human-derived stem cells introduces complex questions about donor consent, withdrawal, and ownership, although these questions are not necessarily unique to HBO research (see, e.g. Holm and Lewis 2022; Sawai et al. 2022; Lewis and Holm 2022, 2024). If a donor’s cells lead to a lucrative discovery, should they have rights to financial benefits or intellectual property claims? Traditional legal frameworks, such as contract and tort law, have had relatively little effect in terms of providing ownership interests to donors. In current practices and regulations, it is common not to consider sharing financial benefits with donors. While there have been disputes over unconsented rights claims, as seen in the Moore v. Regents case (Moore v. Regents of University of California 1990), some consent forms explicitly exclude donors from making any future claims. However, there are signs that a very different legal framework, that of “unjust enrichment”,^7^ may end up altering this situation (Menikoff 2025).^8^

Another area of concern involves intellectual property rights. The ownership of HBOs or their outputs — by researchers, institutions, or corporations — raises ethical and legal questions about the commodification of biological structures that mimic human brain functions (see, e.g. Boers et al. 2019), as well as the proper balance of public and private interests in the ownership, or otherwise, of life and nature.

Further, dual-use concerns highlight the potential for scientific findings to be repurposed for harmful applications, including military technologies. Issues of liability and accountability further complicate the landscape — if experiments with or applications of HBOs yield unintended societal harm or functional capabilities, it remains unclear who would bear responsibility. Liability issues may become particularly important for HBOs integrated with AI and machines, given the current lack of established legal responses to harmful actions such as discriminatory text generation by AI systems (Kataoka et al. 2024a).

Regulatory oversight struggles to keep pace with these developments, with current laws and regulations often failing to apply cleanly to HBO research.^9^ Cross-border inconsistencies in legal standards exacerbate these problems (Lewis and Holm 2024), encouraging the strategic selection of jurisdictions with more permissive regulatory environments. Finally, public perception plays a critical role (see, e.g. Ravn et al. 2023). Research may be legally permissible yet still be perceived as unethical, potentially resulting in lawsuits, damaging the reputations of researchers and institutions, undermining public trust in science, or leading to tighter regulatory oversight. Another worry in this context is over-regulation, i.e. the possibility that an ethically benign research practice is legally prohibited or discouraged (Bublitz 2023). Taken together, these legal challenges demand not only clearer ethical guidelines but also robust, adaptive legal frameworks to ensure that HBO research proceeds responsibly, transparently, and in alignment with public trust.

## Sociocultural Considerations

### Public Understanding of Ethics

While we have emphasized the importance of fostering public understanding of HBO research, equally important is cultivating public understanding of ethics. Novel entities like HBOs would present to some a challenge of “moral confusion”. In a previous discussion of moral status in chimeric animals, Robert and Baylis (2003, 2021) argued that the existence of such beings would introduce inexorable moral confusion in our existing relationships with non-human entities. Their argument suggests that these entities threaten established moral frameworks by disrupting the traditional categorical distinction between entities with partial moral status (like animals) and those with full moral status (like humans). Similarly, the ambiguous moral status of HBOs might add further moral confusion.

People may struggle to maintain their intuitive sense of ethical categories, such as those distinguishing humans from other beings, potentially destabilizing deeply held moral frameworks. However, this challenge can also be reframed as an opportunity. Rather than clinging to intuitive but perhaps unexamined ethical assumptions, the public could be encouraged to engage in deeper reasoning about the principles that confer moral status. For instance, what makes something morally significant: its species membership, its capacity for suffering, its potential for other forms of consciousness, or something else entirely? These foundational questions about moral status arise across various contemporary contexts, from medical care to animal welfare policies to the development of artificial or synthetic biological intelligence systems (Boers et al. 2016; Koplin and Gyngell 2020; Sinnott-Armstrong and Conitzer 2021; Kagan et al. 2023a, b). Engaging with these questions through public discourse and education could strengthen ethical literacy, equipping individuals to think critically about existing and emerging technologies and fostering a more reflective and principled approach to moral decision-making. This deeper ethical engagement is not only relevant to brain organoids but can also enrich public discourse on broader issues at the intersection of science, technology, and society.

### Cultural Variation in Beliefs and Values

In considering the ethical implications of brain organoid research, it is vital to recognize that there is no single, universal way of doing ethics. Different moral frameworks — especially those rooted in non-Western traditions — might approach questions of moral status in ways that differ significantly from dominant Western paradigms. For example, as noted earlier, although Western ethical theories often focus on intrinsic qualities like sentience or other forms of consciousness as the foundation for moral status, some Indigenous and Eastern traditions take a more relational approach, emphasizing an entity’s connections to humans, nature, or other beings. These perspectives view moral worth not as an inherent property but as something arising from an entity’s role in maintaining balance or harmony within a larger network. By incorporating these pluralistic perspectives, discussions about the ethical and legal dimensions of HBO research can become more inclusive, allowing for a richer understanding of how different societies might approach and navigate these novel scientific challenges. In particular, when obtaining consent for cell donation, it is essential to incorporate local cultures to fully respect the beliefs and values of potential donors.

### Need for Empirical Psychosocial Research

Fostering cross-cultural engagement and promoting public understanding of science and ethics must be grounded in careful empirical research into how people actually perceive and understand brain organoids. Lay attitudes, beliefs, and perceptions about organoids — shaped by cultural systems, demographic factors, and individual moral intuitions — can vary widely and may not align with philosophical or scientific frameworks (Evans 2024). For instance, some groups might view organoids primarily through a lens of spiritual or relational ethics, while others might focus on their biomedical potential or risks. Understanding these diverse perspectives through psychosocial and moral psychological research is critical for crafting policies that are not only ethically robust but also socially acceptable and culturally sensitive.

Surveys on people’s perceptions and values regarding HBOs have been increasing in recent years. Research methods are diverse, ranging from semi-structured interviews (Haselager et al. 2020; Bollinger et al. 2021; MacDuffie et al. 2023) and deliberative workshops (Ravn et al. 2023) to questionnaire surveys (Kataoka et al. 2025b; Sawai et al. 2025b; Villanueva et al. 2025), and experimental ethics methods using vignettes (Tanibe et al. 2024; Evans 2024; Ota et al. 2024; for theoretical and methodological background, see Earp et al. 2021; Lewis et al. 2023). The results of these studies are not entirely uniform, but broadly speaking, while many people are generally positive about the study of HBOs, they also have a wide range of ethical concerns with different strengths. From our perspective, particularly interesting findings include that people’s views on nature, politics, religion, or science can influence their evaluation of HBO research (Evans 2024; Villanueva et al. 2025) and that a significant number of people link HBO research to human cloning (Sawai et al. 2025b).

However, one major limitation of the previous studies in this field is the limited participation of non-Western people. The only clear exception is a survey of Japanese citizens’ opinions reported in Sawai et al. (2025b) and Kataoka et al. (2025b). Public attitudes on bioethical issues often depend heavily on cultural and social contexts. For example, in an area closely related to HBO research, US citizens have been observed to be more tolerant than Japanese citizens with regard to human–animal chimera research (Crane et al. 2020). We hope that further research on public attitudes toward HBO research will be conducted across a wide range of regions, including the Asia Pacific, in order to identify and respect potential differences.

Such research would help policymakers identify points of resonance or tension between expert and public views (cf. Lavazza and Chinaia 2023), allowing for more inclusive and effective governance. By systematically studying how different communities conceptualize brain organoids and their ethical implications, we can design policies that reflect a pluralistic understanding of moral status, address public concerns meaningfully, and foster trust and engagement across diverse societal contexts.

## Practical and Policy Recommendations

HBOs are a valuable model with significant potential to advance scientific and medical knowledge. All the relevant parties, including researchers, scientific institutions, research funders, policy makers, research ethics committees, and journal policies, among others, should ensure ethical oversight that adopts a balanced approach that promotes innovation with respect to the many potential benefits of HBOs while addressing potential concerns. For that purpose, we recommend the following based on the considerations outlined above. These recommendations reflect the broad consensus of the Working Group.

Box 1. Recommendations for ethical practices in HBOs research

**Table:** 

1. Ethical discussions based on scientific evidence 2. Anticipatory ethical considerations 3. Contextualized ethical analysis 4. Informed consent framework 5. Ethical consideration for consciousness 6. Developing ethical and regulatory frameworks 7. Managing hype and public perception 8. Interdisciplinary and cross-cultural engagement	

### Ethical Discussions based on Scientific Evidence

Regulators, policymakers, and bioethicists must base discussion of ethical practices in HBO research — including, but not limited to, considerations of the moral status of HBOs — on rigorous scientific evidence. Regulations should be based on the best currently available scientific evidence regarding the structures and capacities of HBOs, while remaining cognizant of scientific uncertainty on these points. Likewise, the basic and translational potential scientific and medical benefits of using HBOs should be considered in such a framework to ensure that a holistic approach is adopted when considering the potential risks and benefits that may arise in the future.

### Anticipatory Ethical Considerations

Researchers, research institutes, policymakers, and other regulatory stakeholders should develop ethical frameworks that are forward-looking to address potential future challenges, such as the emergence of sentience or other complex neural abilities in organoids. This should include consideration of the possible welfare interests of future sentient HBOs and may require taking a precautionary approach informed by proportionality.

### Contextualized Ethical Analysis

Bioethicists and policymakers should assess ethical aspects of HBOs in the broader context of related experimental models, including living or deceased human brains, living or deceased animal brains, human-animal chimeras, human neural cells, and AI systems, to ensure consistency between regulations and comprehensive oversight (Porsdam Mann et al. 2025). In addition to these comparative models, ethical analysis should also take into account extrinsic and relational moral concerns — acknowledging the wider social context and the “situatedness” of HBOs as entities embedded in complex socio-moral networks.

### Informed Consent Framework

Bioethicists, research institutes, and other regulatory stakeholders should develop clear standards for informed consent in HBO research. This includes striking a balance between broad consent for multiple research purposes and specific consent for well-defined purposes. They are also advised to pay significant attention to the design and delivery of information to help donors better understand their participation. International collaboration is also needed to harmonize national and international guidelines to effectively address this challenge.

### Ethical Consideration for Consciousness

It is premature to conclude that HBOs possess sentience or other morally significant forms of consciousness; however, despite this observation, bioethicists and other researchers should not completely rule out such a possibility in future research. Ethical considerations need to address this potential, while emphasizing that the absence of consciousness does not eliminate other concerns.

### Developing Ethical and Regulatory Frameworks

Bioethicists, policymakers, and other regulatory stakeholders should establish robust ethical regulations and guidelines for HBO research. These should be based on best practices in animal experimentation and related fields, while also considering the novelty of sentient HBOs as *sui generis* entities. This requires an ethical approach that should be at least partially original.

For effective regulatory frameworks in this field, a key priority is the harmonization of national and international standards, which is essential for fostering global collaboration. Many regulatory questions remain unresolved. For instance, should the research and use of HBOs be governed by a dedicated parliamentary statute, or would it be better and more appropriate to have non-binding guidelines? How should HBOs be *legally* defined and distinguished from related entities, and what *legal* status should they be assigned? While the global community may work together to address these challenges, reasonable disagreements are likely to arise given the diversity of existing regulatory systems across jurisdictions.^10^ Although the conference included several legal scholars with expertise in their own national contexts, an in-depth discussion of these highly technical regulatory issues was left for future consideration.

### Managing Hype and Public Perception

Researchers, science communicators, media, and policymakers should make every effort to manage public expectations by reducing the hype surrounding HBO research. Clear communication of the scientific realities and limitations of HBOs is essential to avoid sensationalized narratives that can distort ethical and policy discussions and lead to premature and unnecessary regulation.

### Interdisciplinary and Cross-Cultural Engagement

Experts like scientists, ethicists, and policymakers, as well as non-expert individuals from diverse cultural backgrounds, are encouraged to collaborate with each other. This will ensure that diverse perspectives and values are incorporated into the development of ethical and regulatory guidelines for HBO research. An important step to enable this is to create a shared nomenclature that can operate between fields. Collaborative efforts have been initiated through an open call to foster interdisciplinary and cross-cultural engagement (Kagan et al. 2024a, b).

## Conclusion and Future Directions

In this consensus paper, we have provided a high-level overview and a series of ethical recommendations concerning HBO research, based on the intensive deliberations of the Asia Pacific Neuroethics Working Group. Our primary aim was to systematically address longstanding ethical, legal, and sociocultural issues associated with HBO research. The choice to focus primarily on conventional ethical and conceptual debates — such as those surrounding consciousness, moral status, donor consent, and public perception — was deliberate. We acknowledge that our discussion did not extensively incorporate novel applications such as the integration of HBOs with computational or synthetic biological intelligence systems. This limitation is not a shortcoming, but rather reflects the necessity of establishing robust foundational frameworks before adequately addressing the ethical implications of more advanced applications. Indeed, by clarifying and strengthening these foundational ethical principles, we anticipate facilitating informed, nuanced, and contextually sensitive debates about future applications of HBOs, including synthetic biological intelligence.

Furthermore, this paper marks the first effort in the Asia Pacific region to generate a comprehensive set of ethical recommendations specifically focused on HBO research. We believe that such regionally contextualized ethical guidance is critical given cultural variations in ethical values and legal standards. Our intention is to continue this effort, adapting and updating our recommendations in response to evolving scientific developments, ethical insights, and sociocultural perspectives. A key future direction will be deeper engagement with Asia Pacific philosophical traditions that may inform the normative aspects of HBO research. While the authors broadly recognize the importance of integrating both Western and non-Western insights into the ethical discussion, such a task lies beyond the scope of the present consensus paper. Our aim here was to present agreed-upon recommendations — largely shaped by the prevailing Western bioethical framework and existing bioethics literature — rather than to develop novel arguments rooted in Asia–Pacific traditions. Future initiatives will benefit from interdisciplinary and cross-cultural collaboration to ensure that ethical and regulatory frameworks remain responsive to both scientific advances and societal values.

In conclusion, while recognizing our present scope’s boundaries, we emphasize that addressing current ethical questions rigorously is essential groundwork for responsibly navigating the complex ethical terrain associated with future HBO research, including synthetic biological intelligence and other novel applications. We remain committed to ongoing dialogue and engagement to ensure the responsible advancement of HBO research, reflecting the highest ethical standards and broad societal consensus.

## Appendix

On 11–12 November 2024, The International Bioethics Symposium 2024 on “Ethical, Legal, and Social Issues of Human Brain Organoid Research and Application” was hosted at the Kent Ridge Guild House of the National University of Singapore (NUS), Singapore. Invited speakers were Brian D. Earp (National University of Singapore, Singapore), Alexandre Erler (National Yang Ming Chiao Tung University, Taiwan), Tamami Fukushi (Tokyo Online University, Japan), Shu Ishida (Hiroshima University, Japan), Brett J. Kagan (Cortical Labs, Australia), Masanori Kataoka (Hiroshima University, Japan), Julian Koplin (University of Melbourne, Australia), Andrea Lavazza (Pegaso University, Italy), Tsung-Ling Lee (Taipei Medical University, Taiwan), Ilhak Lee (Yonsei University, Korea), Ji Hyun Yang (Yonsei University, Korea), Takuya Niikawa (Kobe University, Japan), Naoya Nagaishi (University of Tokyo, Japan), Masatoshi Kokubo (University of Tokyo, Japan), Eisuke Nakazawa (University of Tokyo, Japan), Koji Ota (University of Tsukuba, Japan), Tsutomu Sawai (Hiroshima University, Japan), and Julian Savulescu (National University of Singapore, Singapore). Affiliations are listed as at the time of the symposium. The Symposium was organized by Professors Tsutomu Sawai and Julian Savulescu and co-sponsored by the Center for Collaborative Sciences of Hiroshima University, Uehiro Division for Applied Ethics at the Graduate School of Humanities and Social Sciences, Hiroshima University, and the Science, Health and Policy-relevant Ethics in Singapore (SHAPES) initiative of the NUS Centre for Biomedical Ethics led by Professor Julian Savulescu. The SHAPES initiative is funded by Singapore Ministry of Health’s National Medical Research Council under its Enablers and Infrastructure Support for Clinical Trials-related Activities Funding Initiative (NMRC Project No. MOH-000951). The purpose of the meeting was to bring together leading voices from the East and the West – including scientists, philosophers, ethicists, and legal scholars – to explore and discuss these critical challenges posed by human brain organoid research and its applications.

## Footnotes

1 However, it is appropriate to say that, at times, researchers in the field themselves have overemphasized their findings (for details, see Vogt et al. 2023).

2 It is important to distinguish between *some* moral status and *full* moral status (i.e., roughly the status of a cognitively normal adult human being). In what follows, when we refer to an entity having or not having moral status, this should be interpreted as “some moral status” unless otherwise specified.

3 Unfortunately, as suggested above, there is a divergence in the use of this word between bioethics and some organoid research (Browning and Birch 2022; Lavazza 2020; Lewis and Holm 2024, 28–33).

4 Another theoretical possibility could be to prohibit research with sentient entities altogether, including sentient research animals and advanced, yet to be developed, HBOs that may display similar sentient traits. We set this possibility aside for the sake of practical considerations, focusing on the comparative question of which is ethically more desirable if we have a choice: to prohibit research with sentient animals or to prohibit HBO research.

5 In addition, several traits that have been understood in individualistic manners, such as agency and autonomy, are increasingly discussed under the relational light. See, for instance, Boers, et al. (2019), Lewis and Holm (2022).

6 Note that the focus on relationships does not necessarily favour the moral status of HBOs. For example, Cordeiro-Rodrigues and Ewuoso (2021) argue against the moral status of HBOs from an Afro-communitarian perspective that emphasizes relationships.

7 An unjust enrichment claim brought against a company by the family of Henrietta Lacks, whose cells were used without consent decades ago to create the first cell line, led that company to settle. And in 2024, another such lawsuit by her family against another company led to a lengthy U.S. federal district court opinion rejecting a motion to dismiss the lawsuit (Lacks v. Ultragenyx Pharmaceutical Inc 2024).

8 Another relevant consideration may be suggested from the Access and Benefit Sharing (ABS) scheme under the Nagoya Protocol (United Nations 2010), which governs access and sharing of (non-human) genetic materials and traditional knowledge between countries. The Protocol provides that benefits are shared with the country where genetic material is derived. If countries can receive benefits from plants and traditional knowledge found in their territories, it seems striking that donors cannot claim benefits (financial or otherwise) derived from their donations once proper consent is obtained.

9 On 29 April 2025, China released the Human Organoid Research Ethical Guidelines, establishing the world’s first national governance framework specifically addressing brain organoids. The Guidelines adopt a preventive governance model that seeks to identify and mitigate ethical risks through pre-research assessment. Key provisions focus on preventing the potential emergence of consciousness by mandating real-time EEG monitoring and implementing complexity caps. Research Ethics Committees are required to include neurobiologists and related experts, while neural data are classified as highly sensitive health information to ensure donor privacy (Zhou et al. 2025).

10 This is not merely a matter of non-Western societies holding different perspectives than Western ethics. Rather, reasonable disagreements may also exist within the Asia–Pacific region itself. For example, in the related field of stem cell–based embryo model (SCBEM) research, each research-intensive country has developed its own regulatory approach to the challenges posed by this emerging technology, reflecting distinct legal traditions and social values (Sawai et al. 2025a).
